# Prevalence and associated factors of chronic kidney disease among adult hypertensive patients in Tigray teaching hospitals: a cross-sectional study

**DOI:** 10.1186/s13104-019-4610-8

**Published:** 2019-09-09

**Authors:** Degena Bahrey, Gebreamlak Gebremedhn, Teklewoini Mariye, Alem Girmay, Woldu Aberhe, Assefa Hika, Girmay Teklay, Hagos Tasew, Teklay Zeru, Hadgu Gerensea, Gebre Teklemariam Demoz

**Affiliations:** 1 Department of Adult Health Nursing, School of Nursing, Aksum University, Aksum, Ethiopia; 2 Department of Anaesthesia, Aksum University, Aksum, Ethiopia; 3 Department of Neonatal Nursing, School of Nursing, Aksum University, Aksum, Ethiopia; 4 Department of Clinical Pharmacy, School of Pharmacy, Aksum University, Aksum, Ethiopia

**Keywords:** Chronic kidney disease, Hypertension, Prevalence, Ethiopia

## Abstract

**Objective:**

The aim of this study was to assess the prevalence of chronic kidney disease and to identify associated factors of chronic kidney disease among hypertensive patients. A cross-sectional study was conducted among selected 578 hypertensive patients. Data were collected using face to face interviewing questionnaires and from medical chart review. Binary logistic regression analyses were performed and analyzed using SPSS version 23.0.

**Result:**

Of the total 578 hypertensive patients the prevalence of chronic kidney disease was found to be 128 (22.1%). Of these hypertensive patients, patients with uncontrolled blood pressure, overweight/obesity, dyslipidemia and diabetic mellitus, 43.3%, 33.7%, 27.3% and 28.2 respectively. After adjustment, the independent variables the significant associated factors of chronic kidney disease among hypertensive patients were age [AOR (95% CI 1.43 (1.07–1.81)], uncontrolled hypertension 4.434 [AOR (95% CI 9.45 (1.34, 14.73)], overweight/obese [AOR (95% CI 7.422 (2.72, 20.28)], dyslipidemia [AOR (95% CI) 13.749 (5.69, 33.215)], diabetic mellitus [AOR (95% CI) 2.137 (1.07, 4.26)]. In conclusion, the prevalence of chronic kidney disease was considerably high. The major associated factors of chronic kidney disease were age, uncontrolled hypertension, overweight/obese, diabetic mellitus and dyslipidemia.

## Introduction

World Health Organization (WHO) report that more than one third (39%) of annual death of Ethiopia population was due to non-communicable disease (NCDs) [1]. High blood pressure also affects almost 1 billion people worldwide and is a leading cause of mortality and morbidity. The future risk of NCD forms of Chronic Kidney Disease (CKD), predominantly driven by increased rates of hypertension, smoking, and obesity, is a growing public health concern [2]. It becomes severe issues for the people living in developing countries [3, 4]. CKD is a progressive failure in renal function for prolonged period of time. Thus, hypertension has been considered as the cause for CKD. Now a day, the prevalence of CKD is increasing worldwide due to an increase in the risk factors such as hypertension, diabetes, hyper-lipidemia, obesity, and smoking [5, 6]. Approximately 6% of patients with essential hypertension have chronic kidney disease (CKD) and are at risk for progression to end-stage renal disease (ESRD). The Seventh Report of the Joint National Committee (JNC 7) and National Kidney Foundation-Dialysis Outcomes Quality Initiative (NKF-DOQI) guidelines identify CKD as a higher-risk category requiring intensive blood pressure control to achieve a blood pressure goal of less than 130/80 mmHg [7].

In Sub Saharan Africa the big alarming is about 32.3% among hypertensive patients have CKD [8].

Investigating the associated factors of CKD among hypertension is very important for prevention and control of CKD. Ethiopia was signed to achieve sustainable development goal (SDG) from 2016 to 2030 and to reduce by one third premature death from non-communicable diseases [9]. However, in Ethiopia little is known related to the prevalence and associated factors of CKD, particularly among hypertensive patients. Therefore, the purpose of this study was to assess the prevalence and associated factors of CKD among adult hypertensive patients attending at Tigray teaching hospitals.

## Main text

### Study setting and study design

A cross-sectional study was conducted in Tigray teaching hospitals from February to April, 2018. The data were collected using face to face interviewing questionnaire and from patients’ medical charts that was recorded from January 1, 2013 to December 30, 2018.

### Study population and sample size determination

All adult hypertensive patients who had hypertension on follow-up care at Tigray teaching hospitals, who met the inclusion criteria were included in the study. The inclusion criteria were hypertensive patients aged 18 years and above, who had regular follow-up in the cardiac clinic. Five hundred seventy eight patients met the inclusion criteria and were included in this study. A questionnaire and checklist was used to collect the data.

### Eligibility criteria

#### Inclusion criteria

All adult hypertensive patients who had treatment follow-up of hypertension at teaching hospitals. Adults aged 18 years or older with hypertension and with e-GFR ≥ 60 mL/min/1.73 m^2^ in January 2013 (this is a baseline assessment) were identified and selected for this study.

#### Exclusion criteria

Hypertensive clients who were pregnant women during the study period and client who had develop the outcome of interest before or at the beginning of follow-up start. Patients with e-GFR < 60 mL/min/1.73 m^2^ were excluded.

### Sampling technique

A consecutive sampling technique was employed, and every patient was included in the study.

### Data collection instrument and techniques

Data was collected using a semi-structured face to face interviewing questionnaire and patients chart review. The questionnaire had two subparts, namely socio-demographic and clinical characteristic. Te questionnaire was initially prepared in English then translated into the local language (Tigrigna) by an individual who has good ability of the two languages then translated back to English by different person to ensure consistency. A week prior to the actual data collection period, the questionnaire was pre-tested on 5% of the total sample size patients identified from Mekelle Hospital. Data was collected by four trained B.Sc. nurses and two supervisors (B.Sc. nurses).

### Measurement tools

*Urine dipstick test* Patient was asked to submit random midstream urine sample prior to admission in a 50 mL urine container for laboratory analysis for random urine dipstick test, protein, and creatinine. The dipstick analysis was done using the uriplus 900 urinalysis stip. The following are the grades of proteinuria as provided by the manufacturers:

- 0: absent
- Traces: 15 to 30 mg/dL
- 1+: 30 to 100 mg/dL
- 2+: 100 to 300 mg/dL
- 3+: 300 to 1000 mg/dL
- 4+: greater than 1000 mg/dL.

*Estimated GFR* is a test that is used to assess how well kidneys are working. The test estimates the volume of blood that is filtered by kidneys over a given period of time. It can be measured by using readily available equations and formulas like Cockcroft–Gault. CockcroftGault formula (normalized for the body surface area [BSA]): (140 − Age [years]) × weight (kg) × (0.86, if female) × 1.73/72 × serum creatinine (mg/dL) × BSA (m^2^). In this case BSA will be calculated by using Mosteller formula that is BSA is equal to $\sqrt{W\,*\,H}$ divided by 60, while weight is measured in kilogram and height in centimetre.

### Data processing and analysis

Data was coded, entered, edited, and cleaned by Epi-data manager version 4.2 and then exported into SPSS version 23 for analysis. Chronic kidney disease was confirmed by reviewing medical chart in the hospitals. Baseline demographic, clinical, laboratory and social characteristics were used as independent variables in the analysis.

Binary logistic regression model was use to infer the association between the outcome and independent variables. In the bi-variates analysis variables with P-value < 0.25 was included in the multivariable binary logistic regression. Odds ratio with 95% confidence level was computed and P-value < 0.05 was described as having a significant association.

### Study variables

#### Dependent variable

Chronic kidney disease.

#### Independent variable

*Socio-demographic factors* Age, sex, religion, educational, occupational, income and marital status.

*Clinical characteristic* Blood pressure status, baseline diastolic blood pressure, baseline systolic blood pressure and baseline, stage of hypertension, type of hypertension, number of drugs, duration on drug, types of drugs, diabetes mellitus, dyslipidemia and obese.

### Operational definitions

*Chronic kidney disease* for this study we consider patient having CKD if the physician was diagnosed as having CKD and documented in the patient’s medical chart. Chronic Kidney disease is defined as e-GFR < 60 mL/min/1.73 m by Cockcroft Gault equations.

*Controlled blood pressure* BP < 140/90 mmHg using digital sphygmomanometer for adult hypertensive clients without diabetes mellitus and chronic kidney disease for at least three consecutive follow-up measurement and blood pressure < 130/80 mmHg using digital sphygmomanometer for adult hypertensive clients with diabetes mellitus and chronic kidney disease for at least three consecutive follow-up measurement.

*BMI (body mass index)* A measure of the person’s weight in kilogram divided by his height in meter square. Based on this calculation body weight classified as underweight for patient with less than 18.5 kg/m^2^, normal weight 18.5–24.9 kg/m^2^, overweight 25–29.9 kg/m^2^ and obese for patients with BMI ≥ 30 kg/m^2^ [10].

*Dyslipidemia* if the patients having either of the following [10]: total cholesterol ≥ 200 or triglyceride 150 mg/dL (1.7 mmol/L) or specific treatment for this lipid abnormality or High density lipoprotein < 40 mg/dL (1.03 mmol/L) in male < 50 mg/dL (1.29 mmol/L) in female or specific treatment for this lipid abnormality.

*Proteinuria* according the laboratory result if a patient had plus one and above we classify as having proteinuria.

*Diabetic mellitus* was defined as self-reported diabetes or the use of hypoglycaemic agents or both.

*Hypertension* was defined as those who had a documented diagnosis of hypertension (i.e. BP ≥ 140/90 mmHg) or those on anti-hypertensive agents. Target control BP was defined as BP < 140/90 mmHg for non-diabetic and < 130/80 mmHg for diabetic patients.

## Results

### Socio-demographic characteristics

Five hundred seventy eight hypertensive patients were fulfilling the inclusion criteria and included in this study. Out of the total patients half, 290 (50.2%), were females. The documented age of the patients ranges from 30 to 87 years with the median age of 54.14 years. From the patients more than half 323 (55.9%). were urban residing. Out of the total patients almost all were Christian orthodox in religion 539 (93.3%) (Table 1).

**Table 1:** Socio-demographic characteristics among adult hypertensive patients attending at Tigray teaching hospitals, Ethiopia, 2018 (n = 578)

Variables	Category	Frequency	Percept
Sex	Male	288	49.8
	Female	290	50.2
Marital status	Married	498	86.2
	Single	35	6.1
	Widow	34	5.9
	Divorced	11	1.9
Religion	Christian orthodox	539	93.3
	Muslim	38	6.6
Ethnicity	Tigray	556	96.2
	Afar	13	2.3
	Amhara	9	1.5
Residence	Urban	323	55.9
	Rural	255	44.1

### Clinical characteristics

Among the total participants patient nearly one quarter 128 (22.1%) were patient with CKD. 163 (28.2%) diabetic mellitus and 158 (27.3) dyslipidemia. Based on medical condition and life style risk of hypertensive patients were common in patient co-morbid with DM, dyslipidemia and patient with Proteinuria.

Among patients with chronic kidney disease 96% patients were having uncontrolled hypertension, 91.2% patients were overweight /obese, 81.2% were patients with dyslipidemia, 81.2% were patients in stage three and above hypertension, 75%, were patients greater than 60 years old, 74.2% were patient lived in urban and 60% patients were with DM (Table 2).

**Table 2:** Clinical characteristics among adult hypertensive patients attending at Tigray teaching hospitals, Ethiopia, 2018 (n = 578)

Variables	Category	Frequency	Percent
Type of hypertension (HTN)	Systolic HTN	93	16.1
	Mixed	485	83.9
Classification of HTN	Primary	555	96
	Secondary	23	4
Number drug	One	343	59.3
	Two	218	37.7
	Three and above	17	2.9
Proteinuria	Yes	146	25.3
	No	432	74.7
Chronic kidney disease	Yes	128	22.1
	No	450	77.9
Diabetic mellitus	Yes	163	28.2
	No	415	71.8
Dyslipidemia	Yes	158	27.3
	No	420	72.7
Blood pressure status	Controlled	328	56.7
	Uncontrolled	250	43.3
Body mass index	Normal	383	66.3
	Over weight/obese	195	33.7

## Discussion

This study was tried to assess the prevalence and associated factors of chronic kidney disease among hypertensive patients. In this study, the socio-demographic and clinical characteristics of 578 patients were analysed. From the present study, we found that approximately one quarter of the patients or one in five patients’ 128 (22.1%) with 95% CI (18.5–25.4) were had an evidence of chronic kidney disease using the calculated e-GFR. This result was lower than the study done in Malaysia [11], Ghana [12], in Sub-Saharan Africa [8], Democratic Republic of Congo [13] and Nigeria [14] (31%), 47%, 32.3%, 36% and 55.49% respectively. This discrepancy may be due the difference in study design, study population, and age of the participants.

On the other hand our study was higher than with study conducted in US (6%) [15]. The variation may be due standard living condition of the two countries. Our study also higher than with study conducted in Ethiopia that was (12.2%) [16]. The difference may be due the study design and difference in study population.

The national chronic kidney disease fact sheet of 2017 showed among hypertensive patients one from five patients have chronic kidney disease which is in line with our study [17]. This similarity might be due the science hypertension is the risk factor of CKD. From the multivariable logistic regression age, uncontrolled hypertension, overweight and obese and dyslipidemia were the associated variable to develop chronic kidney disease in hypertensive patients.

In this study the associated factors of chronic kidney disease were age with greater than 60 years [AOR (95% CI 1.43 (1.07–1.81)], which is in line with study conducted in Malaysia, Cameroon, China, Ghana, Democratic Republic of Congo and Nigeria [8, 11–15, 18, 19]. This might be due to the nature as the age increase, the function of the kidney decrease. overweight and obese [AOR (95% CI 7.422 (2.72, 20.28)], uncontrolled hypertension 4.434 [AOR (95% CI 9.45 (1.34, 14.73)], dyslipidemia [AOR (95% CI) 13.749 (5.69, 33.215)], diabetic mellitus [AOR (95% CI) 2.137 (1.07, 4.26)] which is in line with study conducted in Malaysia, Cameroon, China, Ghana, Democratic Republic of Congo and Nigeria [8, 11–15, 18, 19] (Table 3).

**Table 3:** Bivariate and multivariable logistic regression analysis of chronic kidney disease among adult hypertensive patients attending at Tigray teaching hospitals, Ethiopia, 2018 (n = 578)

Variables	Category	Bivariate				Multivariable			
		P-value	COR	95.0% CI		P-value	AOR	95.0% CI	
				Lower	Upper			Lower	Upper
Blood pressure	Controlled (Ref)	1	1	1	1	1	1	1	1
	Uncontrolled	0.001*	7.76	5.29	11.38	0.02*	4.43	1.34	14.73
Age	≥ 60 years	0.001*	6.67	4.58	19.70	0.02*	1.43	1.07	1.81
	< 60 years (Ref)	1	1	1	1	1	1	1	1
Proteinuria	Yes	0.001*	183.59	65.79	512.24	0.06	5.068	1.93	27.43
	No (Ref)	1	1	1	1	1	1	1	1
BMI	Over weight /obese	0.001*	32.69	20.27	52.73	0.01*	9.450	4.92	18.15
	Normal (Ref)	1	1	1	1	1	1	1	1
Dyslipidemia	Yes	0.001*	11.26	7.31	17.34	0.01*	13.75	5.69	33.22
	No (Ref)	1	1	1	1	1	1	1	1
DM	Yes	0.001*	5.01	3.40	7.38	0.03*	2.137	1.07	4.26
	No (Ref)	1	1	1	1	1	1	1	1
Residence	Urban	0.642	1.256	0.480	3.287	0.99	1.05	0.80	4.2
	Rural (Ref)	1	1	1	1	1	1	1	1

*Ref* reference category, *COR* crude odds ratio, *AOR* adjusted odds ratio, *BMI* body mass index, *DM* diabetic mellitus

* Variables with significant association

## Conclusion

In this study the prevalence of chronic kidney disease among hypertensive patients were considerably high. Age, overweight, uncontrolled hypertension and dyslipidemia were found to be associated factors of CKD. In response to this finding, tailored future intervention that targets in prevalence and resolution of associated factors is required.

## Limitations

Limitation of our study were excluding of incomplete medical chart and this study, we did not consider the renal biopsy for the diagnosis of CKD.

## Data Availability

The datasets used and/or analysed during the current study are presented within the manuscript and available from the corresponding author on reasonable request.

## Abbreviations

BMI: body mass index
BP: blood pressure
CKD: chronic kidney disease
CVD: cardiovascular disease
DM: diabetic mellitus
NCD: non-communicable disease
